# NMR‐identification of the interaction between BRCA1 and the intrinsically disordered monomer of the Myc‐associated factor X

**DOI:** 10.1002/pro.4849

**Published:** 2024-01-01

**Authors:** Ludovica Martina Epasto, Christopher Pötzl, Herwig Peterlik, Mahdi Khalil, Christine Saint‐Pierre, Didier Gasparutto, Giuseppe Sicoli, Dennis Kurzbach

**Affiliations:** ^1^ Faculty of Chemistry, Institute for Biological Chemistry University of Vienna Vienna Austria; ^2^ Vienna Doctoral School in Chemistry (DoSChem) University of Vienna Vienna Austria; ^3^ Faculty of Physics University of Vienna Vienna Austria; ^4^ CNRS UMR 8516, LASIRE University of Lille Villeneuve d'Ascq Cedex France; ^5^ Universite Grenoble Alpes Grenoble France

**Keywords:** complexation, MAX, NMR, RCA1

## Abstract

The breast cancer susceptibility 1 (BRCA1) protein plays a pivotal role in modulating the transcriptional activity of the vital intrinsically disordered transcription factor MYC. In this regard, mutations of BRCA1 and interruption of its regulatory activity are related to hereditary breast and ovarian cancer (HBOC). Interestingly, so far, MYC's main dimerization partner MAX (MYC‐associated factor X) has not been found to bind BRCA1 despite a high sequence similarity between both oncoproteins. Herein, we show that a potential reason for this discrepancy is the heterogeneous conformational space of MAX, which encloses a well‐documented folded coiled‐coil homodimer as well as a less common intrinsically disordered monomer state—contrary to MYC, which exists mostly as intrinsically disordered protein in the absence of any binding partner. We show that when the intrinsically disordered state of MAX is artificially overpopulated, the binding of MAX to BRCA1 can readily be observed. We characterize this interaction by nuclear magnetic resonance (NMR) spectroscopy chemical shift and relaxation measurements, complemented with ITC and SAXS data. Our results suggest that BRCA1 directly binds the MAX monomer to form a disordered complex. Though probed herein under biomimetic in‐vitro conditions, this finding can potentially stimulate new perspectives on the regulatory network around BRCA1 and its involvement in MYC:MAX regulation.

Transcription factors (TFs) play an essential role in many biological processes, such as cell cycle regulation (Amati & Land, 1994; Theilgaard‐Mönch et al., 2022; Yang et al., 2007) and cell replication (Helin, 1998), for which high structural plasticity is often required (Brodsky et al., 2020; Lambert et al., 2018). Particularly, cancer‐associated TFs often feature intrinsically disordered regions (IDRs) (Bushweller, 2019; Liu et al., 2006). An ubiquitous example of an intrinsically disordered TF is the basic helix–loop–helix leucine zipper (bHLH‐LZ) MAX, which adopts a characteristic homodimer structure. Each dimer unit consists of two helices separated by a loop (HLH domain), flanked by the intrinsically disordered basic N‐terminus and a C‐terminal leucine zipper (LZ) domain (Sammak et al., 2019; Sauvé et al., 2004). The MAX conformational space is quite heterogeneous: The homodimer exists in a conformational equilibrium with a set of dissociated intrinsically disordered monomer states (Fieber et al., 2001; Turner, 2003). In order to form a transcriptionally active complex, MAX can heterodimerize with several interaction partners, such as MYC (Kretzner et al., 1992; Lavigne et al., 1998), Mad (Ayer et al., 1993; Nair & Burley, 2003), Mxi1 (Zervos et al., 1993). In particular, the MYC:MAX heterodimer has been intensively studied (Amati et al., 1993; Ecevit et al., 2010; Epasto et al., 2022; Hu et al., 2005; Kizilsavas et al., 2017; Kretzner et al., 1992; Macek et al., 2018; Panova et al., 2019; Sammak et al., 2019; Turner, 2003; Wechsler et al., 1994).

BRCA1 (breast cancer susceptibility protein 1) (Petrucelli et al., 2010; Venkitaraman, 2002), a large partially disordered protein containing 1863 amino acids, is an established key player in regulating MYC:MAX (Grushko et al., 2004; Ren et al., 2013; Wang et al., 1998). BRCA1 mainly expresses its modulatory effect through its IDR, spanning amino acids ~200 to ~500. A dysregulation of BRCA1 expression is linked to breast and ovarian cancer syndrome outbreak (HBOC) (Grushko et al., 2004; Petrucelli et al., 2010).

The interaction between MYC and BRCA1 is well established (Wang et al., 1998), in contrast to the binding of BRCA1 to MAX. The latter has surprisingly not been observed so far, despite a high primary sequence similarity between both proto‐oncoproteins (Amati & Land, 1994; Fieber et al., 2001; Kretzner et al., 1992; Turner, 2003). Herein, we complement the current conception of the BRCA1 interaction network by showing that BRCA1 indeed does bind MAX, yet only when in its less common intrinsically disordered monomeric state (Kizilsavas et al., 2017). In such a conformation, the MAX monomer indeed features structural characteristics similar to the MYC monomer, enabling its BRCA1 interaction. The by residue‐resolved NMR (nuclear magnetic resonance), SAXS (small‐angle x‐ray scattering), and ITC (isothermal titration calorimetry) that MAX monomers form dynamic, fuzzy complexes (Fuxreiter & Tompa, 2012) with BRCA1.

To probe the MAX‐BRCA1 interaction, we selected a BRCA1 fragment housing the amino acids 219–504 (denoted henceforth as BRCA1^219‐504^) involved in the binding of c‐MYC's C‐terminus (Wang et al., 1998). We first recorded residue‐resolved NMR signal amplitude changes and ^1^H‐^15^N chemical shift perturbations (CSP, Figure 1). We recorded our experiments in an environment (Kizilsavas et al., 2017) that mimics that found close to the DNA in a cell nucleus to approach near‐physiological conditions, that is, pH 5.5, high organic salt concentrations (see the SI for details).

**FIGURE 1:**
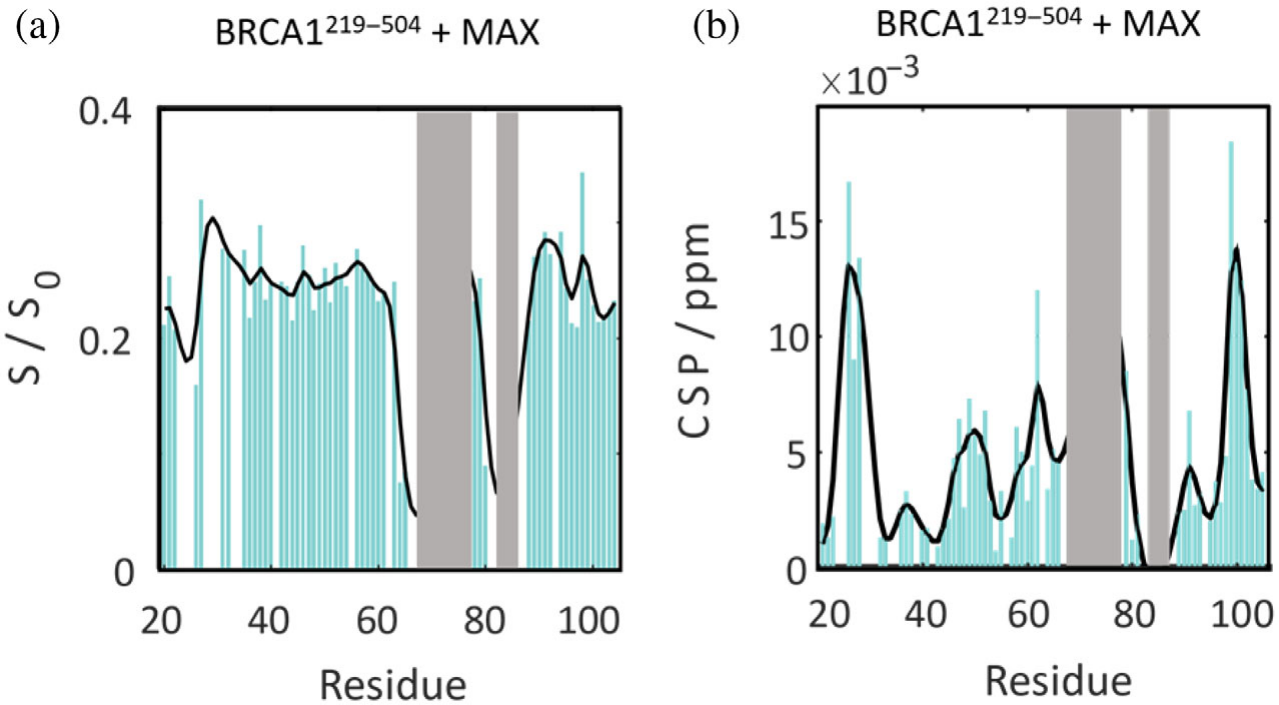
Signal intensity ratios S/S_0_ (left) and CSP (right) for MAX monomers upon binding to BRCA1^219‐504^. The black lines guide the eyes, and the gray bars indicate areas that could not be unambiguously assigned.

First, we exposed BRCA1^219‐504^ to the well‐known MAX:MAX homodimer. No significant changes in the spectra could be observed, which is also in line with literature reports (Wang et al., 1998). The picture drastically changed when elevating the sample temperature to 35°C. At the experimental pH of 5.5, the intrinsically disordered form of MAX dominates its conformational space at this temperature (Fieber et al., 2001; Kizilsavas et al., 2017).

Note that at lower pH, the interaction network constituted of hydrogen bonds and electrostatic interactions between the two MAX monomer units, in particular, the helical and leucine zipper domains, is altered and, hence, interrupted (see, e.g., Fieber et al., 2001). Thus, the monomeric state is favored. At neutral pH the anchor points between the two subunits yet remain intact favoring the dimer (Fieber et al., 2001).

Under our experimental conditions that, thus, favor population of the MAX monomer, the presence of BRCA1^219‐504^ significantly reduced MAX's NMR signal amplitudes *S* relative to those of free MAX *S*_0_ along the entire primary sequence (Figure 1a). The reduced amplitudes point towards reduced tumbling of the entire IDP upon MAX‐BRCA1^219‐504^ complex formation. The high molecular weight of the BRCA1‐MAX assembly (34.5 + 10 KDa) can readily account for the observed amplitude losses. Furthermore, chemical shift perturbations (CSP) with varying intensity were recorded along the entire primary sequence (Figure 1b), corroborating the interaction. The heterogeneous nature of the amplitude changes and CSP indicates a complex binding mechanism between the two IDPs that involves a large part of MAX. Most importantly, though, the data show that BRCA1^219‐504^ interacted with the MAX monomer—contrary to MAX:MAX—which is not unexpected given its sequence homology with c‐MYC (Fieber et al., 2001).

Note that the CSP were significant yet relatively weak, <0.02 ppm, which indicates that neither IDPs underwent strong structural adaptions upon exposure to BRCA1^219‐504^. Instead, the formation of a fuzzy complex, without a specific 3D structure appears more likely.

The most prominent CSP were observed for residues 25–30 and around residue 100. Less intense yet still significant CSP were observed for residues 40–50 and around position 60. These regions are particularly rich in basic amino acids, such as R^25^, H^27^ and H^28^ or K^89^ and R^90^. Hence, potentially, the driving force behind the interaction between MAX and BRCA1^219‐504^ is based on hydrogen bonds and/or electrostatic attraction constituted by protonated basic side chains. ITC data (vide infra) confirmed that the nature of the interaction is enthalpically driven and, hence, supports a hydrogen bond or electrostatically driven binding mode.

From the perspective of BRCA1^219‐504^, residues 400–430 showed strong CSP (see Figure S1). This stretch houses 11 acidic amino acids (D or E), which aligns well with the above assumption of basic MAX residues as underlying interaction hot spots.

To probe the source of the amplitude reduction observed in Figure 1, we recorded ^15^N transverse relaxation times *T*_2_, and heteronuclear ^1^H‐^15^N Overhauser enhancements η. We observed heterogeneous changes in both *T*_2_ and η upon complex formation along the entire primary sequence (Figure 2a,b). This again corroborates the “fuzzy” (Fuxreiter & Tompa, 2012) binding mode. The 1/*T*_2_ and η values both grew significantly, showing that the formed complex experiences much‐reduced dynamics compared to the free IDP in solution. We observed a tendency towards particularly large Δ*T*_2_ and Δη values within the HLH domain (residues 30–50) and the N‐terminal part of the IDP (residues 90–104).

**FIGURE 2:**
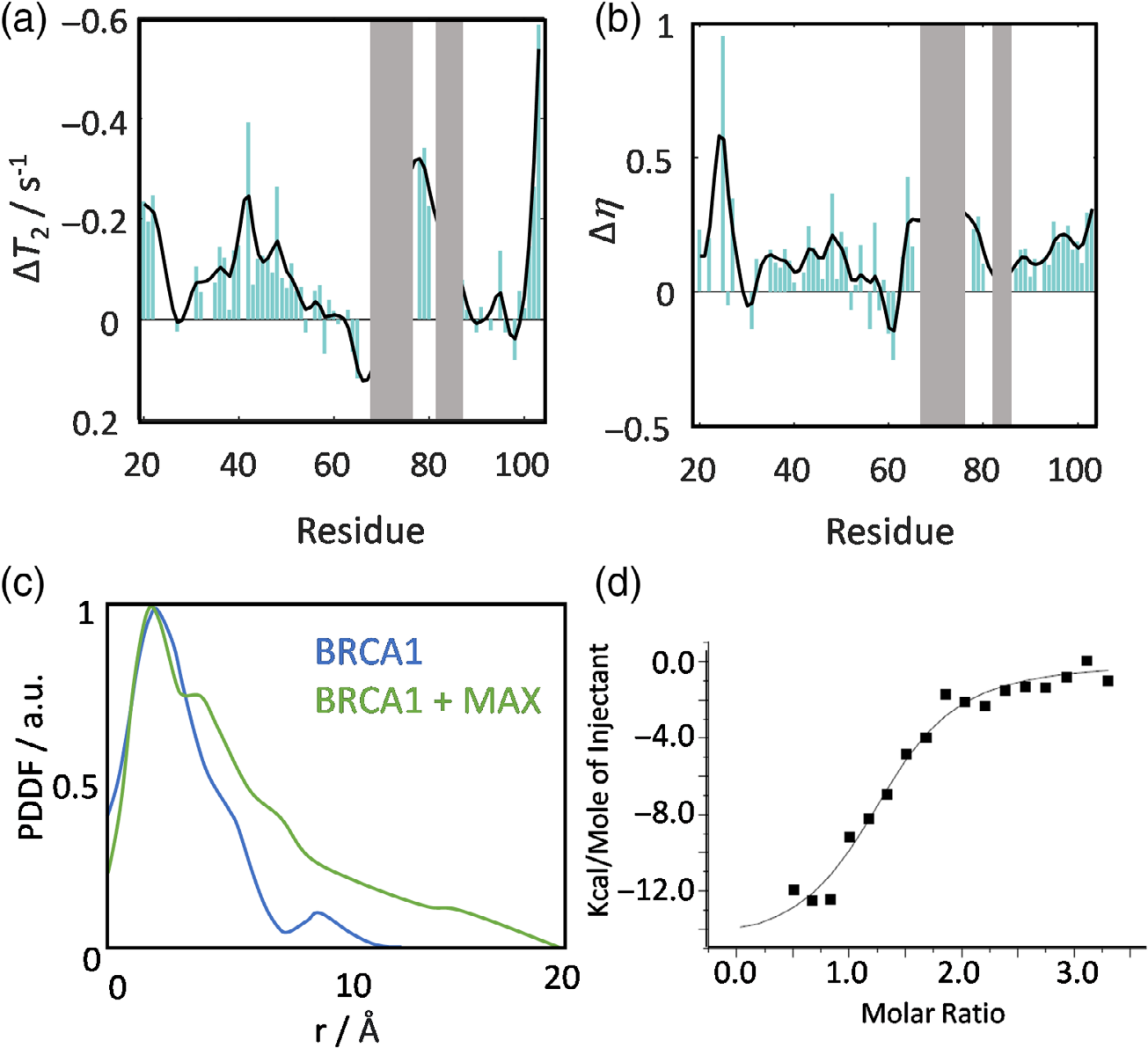
(a, b) Differences in transverse relaxation times Δ*T*_2_ and NOE Δη for MAX monomers upon binding to BRCA1^219‐504^. (c) SAXS PDDF of BRCA1^219‐504^ in the absence (blue) and presence of MAX (green). (d) ITC profile upon titration of MAX into a BRCA1^219‐504^ solution.

Overall, the resulting amplitude loss (Figure 1) observed for MAX aligns well with reduced dynamics, resulting in faster transverse relaxation and line broadening (see the Figure S1–S6 for supplementary relaxation data and NMR spectra). It should furthermore be noted that a contribution to the observed line broadening based on a chemical exchange between the BRCA1^*219‐504*^‐bound and ‐free forms cannot be excluded based on the recorded relaxation parameters.

To corroborate the formerly undocumented interaction between the MAX monomer and BRCA1, we conducted ITC and SAXS experiments (Figure 2c,d). ITC showed a titration profile that could be fitted to a *K*_D_ of 2.5 ± 0.6 μM and a stoichiometry of ca. 1:1.3 ± 0.06 using a one‐site model (see the Figure S7 and S8 for all fit parameters and negative control experiments).

In agreement, SAXS led to a clear increase in atom‐to‐atom distances upon addition of BRCA1 to MAX again corroborating the interaction of the MAX monomer with BRCA1. The pair distance distribution functions (PDDF), extracted from SAXS intensity curves, show an asymmetric profile, typical for intrinsically disordered proteins, with only one maximum for BRCA1^219‐504^, but two maxima for mixtures with MAX indicating the formation of a joint complex.

Overall, the combined NMR, ITC and SAXS data indicate that BRCA1 interacts with MAX monomers in a complex manner under the conditions probed herein. On the one hand, this finding is unexpected as the MAX:MAX dimer does not bind to BRCA1 (Wang et al., 1998)—a fact that can be considered as a paradigm of TF activity. On the other hand, the high sequence similarity between the two fully disordered proteins MYC and MAX in their monomeric states readily rationalizes the observed interaction.

Even though our in‐vitro NMR conditions are not identical to intracellular environments, our results may provide new perspectives to approach BRCA1 and the MYC:MAX interaction network—one intensively researched anti‐cancer drug target (Dauch et al., 2016; Dubiella et al., 2021; Venkitaraman, 2002). For example, the sequestrating of the MAX monomer by BRCA1 to inhibit MYC arises as a possible interaction mechanism within the BRCA1 interaction network.

## AUTHOR CONTRIBUTIONS

**Dennis Kurzbach:** Conceptualization; validation; project administration; supervision; writing – original draft; funding acquisition. **Ludovica Martina Epasto:** Investigation; formal analysis; writing – review and editing. **Christopher Pötzl:** Investigation; formal analysis; writing – review and editing. **Herwig Peterlik:** Investigation; formal analysis; writing – review and editing. **Mahdi Khalil:** Investigation; formal analysis; writing – review and editing. **Christine Saint‐Pierre:** Investigation; formal analysis; writing – review and editing. **Didier Gasparutto:** Conceptualization; investigation; writing – review and editing. **Giuseppe Sicoli:** Conceptualization; investigation; supervision; writing – review and editing.

## CONFLICT OF INTEREST STATEMENT

The authors declare no conflicts of interest.

## Supporting information


**Data S1:** Supporting Information.

## Data Availability

All data are available under https://phaidra.univie.ac.at/o:2037711.
