# Supplementary material for: NMR‐identification of the interaction between BRCA1 and the intrinsically disordered monomer of the Myc‐associated factor X
Source: Protein Sci. 2024 Jan 1;33(1):e4849. doi: 10.1002/pro.4849 (PMC10731500; doi:10.1002/pro.4849)
Supplement: Supplementary file 1 — Data S1: Supporting Information. [file PRO-33-e4849-s001.docx]

**Supporting information**

**Materials and methods**

*Protein expression and sample preparation*

The his-tagged MAX, and BRCA1 were expressed in Rosetta 2 *E. coli* cells, transformed with pET21(a)+ vectors subcloned with the codifying DNA sequences of interest. The bacterial cultures were grown at 37° C until reaching an absorbance of 0.6 at 600 nm. Optionally, the cells were resuspended in M9 (with homogeneously ^13^C labeled glucose 1 g/L and ^15^N ammonium chloride 1 g/L) before induction through IPTG, to achieve a ^13^C or ^15^N homogeneous labeling. The expression was carried out overnight at 30° C. To express the unlabeled protein, the cells were grown in 2YT medium and directly induced by IPTG. They were then grown overnight at 37° C. For the extraction and purification of BRCA1 and MAX we followed the procedures reported in ^1^ and ^2^, respectively. The cells were disrupted, and the insoluble fraction was resuspended in denaturing buffer (25 mM Tris, 100 mM NaCl, 6 M GuCl, pH 8.0) overnight. The proteins were then purified with affinity chromatography. The his-tag was cleaved using TEV protease. The proteins were finally transferred into the MES buffer (25 mM NaCl, 25 mM MES, 100 mM ArgHCl, pH 5.5) using centricons with a cutoff molecular weight of 3 000 Da.

We conducted our measurements at a temperature of 35° C and pH 5.5 to populate the monomeric state by >90%.^3^ For the experiments, we chose a buffer containing 25 mM MES, 25 mM NaCl, and 100 mM ArgHCl at pH 5.5 in order to mimic the cellular environment in the proximity of chromatin.

*NMR*

Nuclear magnetic resonance (NMR) spectra were acquired at 35° C, employing Varian Direct Drive 600 or 800 MHz spectrometers. The data collection was conducted in the States-TPPI/PFG sensitivity-enhanced mode, employing quadrature detection. The carrier frequencies for ^1^H and ^15^N were set at 4.73 and 120.0 ppm, respectively. The sample compositions comprised 1.2 mM MAX, 25 mM MES, and 25 mM NaCl, adjusted to a pH of 5.5, dissolved in a solvent mixture of 90% H_2_O and 10% D_2_O. Typical ^1^H and ^15^N pulse lengths were 8 and 30 μs, respectively.

Subsequently, the NMR spectra were processed and analyzed using a combination of NMRPipe^4^, SPARKY^5^, and custom scripts developed in MATLAB. Apodization was applied using a squared and 60° phase-shifted sine bell window function in all dimensions. The time domain data were zero-filled to twice the original data set size before undergoing Fourier transformation. The pulse sequences utilized in data acquisition were designed to enhance sensitivity, employing gradients for coherence selection and water suppression.

CSP values were calculated as a combination of differential ^1^H and ^15^N chemical shifts Δδ(^1^H) and Δδ(^15^N):

CSP = lΔδ(^1^H)l + lΔδ(^15^N)l ⋅ γ(^15^N)/γ(^1^H) (1)

With γ(^15^N) and γ(^1^H) being the respective gyromagnetic ratios.

For *R*_2_ evaluation, the data were also collected at an 800 MHz proton Larmor frequency. Relaxation delays of 0, 16, 32, 64, 128, 192, and 256 ms were used, with a recycling delay of 1 second. The decay of cross-peak intensities in the series of spectra was fitted to a two-parameter exponential decay model:

*I*(*t*) = A * exp(-τ/*T*_2_) (2)

where *I*(*t*) represents the peak intensity, and τ is the delay time.

Heteronuclear steady-state NOE ^15^N{^1^H^N^} attenuation factors were determined at 14.1 T by calculating the η ratio, which is defined as η = I_NOE_ / I_REF_, where I_NOE_ and I_REF_ represent the peak amplitudes in experiments with and without proton presaturation. For spectra without saturation, a net relaxation delay of 5 seconds was applied, while for NOE spectra, a relaxation delay of 2 seconds was followed by a 3-second proton presaturation period.

The acquisition parameters used were identical to those employed in the PFG sensitivity-enhanced 2D ^1^H−^15^N HSQC experiments.

The signal assignment can be found in references ^6-7^. All further details about the NMR experiments can be found in the provided raw data set (see the Data Availability section).

*ITC*

Titration calorimetric measurements were carried out using a Microcal iTC200 calorimeter. The product of the initial ligand concentration and the association constant c = K_b_ ⋅ [Max] was in the range of 2 < c < 200. The starting conditions were 130-150 μM BRCA1 in the measurement cell (200 μL) and 0.3 mM of MAX in the injection needle (40 μL at pH 5.5 where the monomer state dominates the conformational space). Titration of MAX into buffer gave some heat release. These titrations were subtracted from runs performed with BRCA1. Measurements were carried out with a stirring speed of 1000 rpm. The time delay between the injections was set to 180 s to allow peak recovery to baseline. Nonlinear least-squares fits (one binding site) were employed to interpret the experimental data.

*SAXS*

SAXS scattering patterns were collected using X-rays either from a microfocus source (Incoatec IµS High Brilliance) or a liquid metal jet technology (Excillum) and a 2D position sensitive detector (Vantec 2000, Bruker AXS). The sample-to-detector distance was chosen to cover a range of scattering vector q from 0.1 to 2.8 nm^-1^. All SAXS patterns were radially averaged to obtain scattering intensities depending on the scattering vector q = 4π(sinθ)/λ where 2θ is the scattering angle and λ = 0.1542 nm is the X-ray wavelength in the case of the microfocus source and λ = 0.13414 nm for the metal jet source. The SAXS data are analyzed by the indirect Fourier transformation (IFT) method using the GIFT software.^8^ The resulting pair distance distribution function (PDDF), extracted model-free from the scattering pattern, provided information on the structure of the scattering objects. The MAX and BRCA1 concentrations were 0.1 mM, again at pH 5.5 to overpopulate the MAX monomer.

**Supplementary Data**

**Figure S1**. CSP for BRCA1^219-504^ upon binding to MAX.

**Figure S2**. ^15^N *T*_2_ times for the MAX monomer. The grey areas indicate non-assigned residues.

**Figure S3**. ^15^N *T*_2_ times for the MAX monomer in the presence of BRCA1^219-504^. The grey areas indicate non-assigned residues.

**Figure S4**. ^1^H{^15^N} NOE for the MAX monomer. The grey areas indicate non-assigned residues.

**Figure S5**. ^1^H{^15^N} NOE for the MAX monomer in the presence of BRCA1^219-504^. The grey areas indicate non-assigned residues.

*
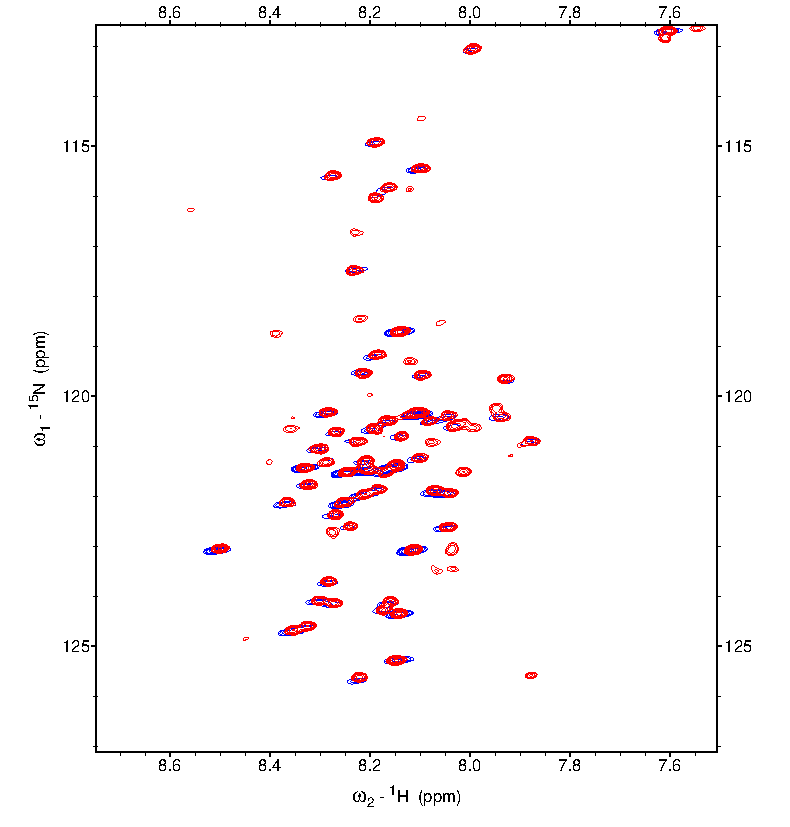

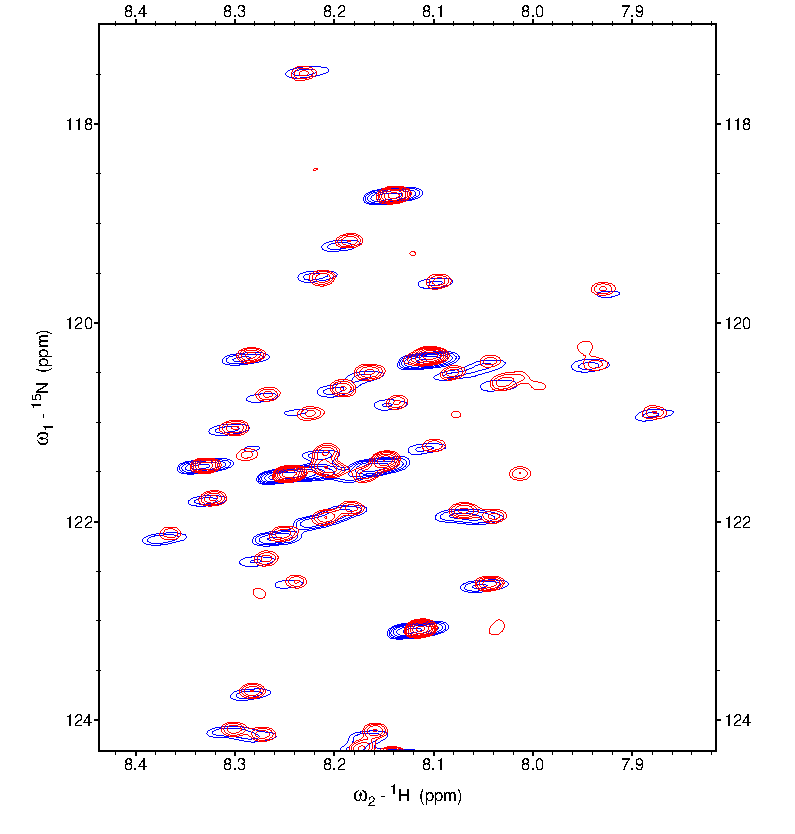
*

**Figure S6**. Comparison of ^1^H-^15^N HSQC of MAX recorded at 35°C and pH 5.5 (blue spectrum). Under these conditions, the monomer dominates the conformational space leading to low chemical shift dispersion in the direct dimension. The presence of BRCA1^219-504^ (red spectrum) led to slight yet significant changes throughout the entire spectrum.

**
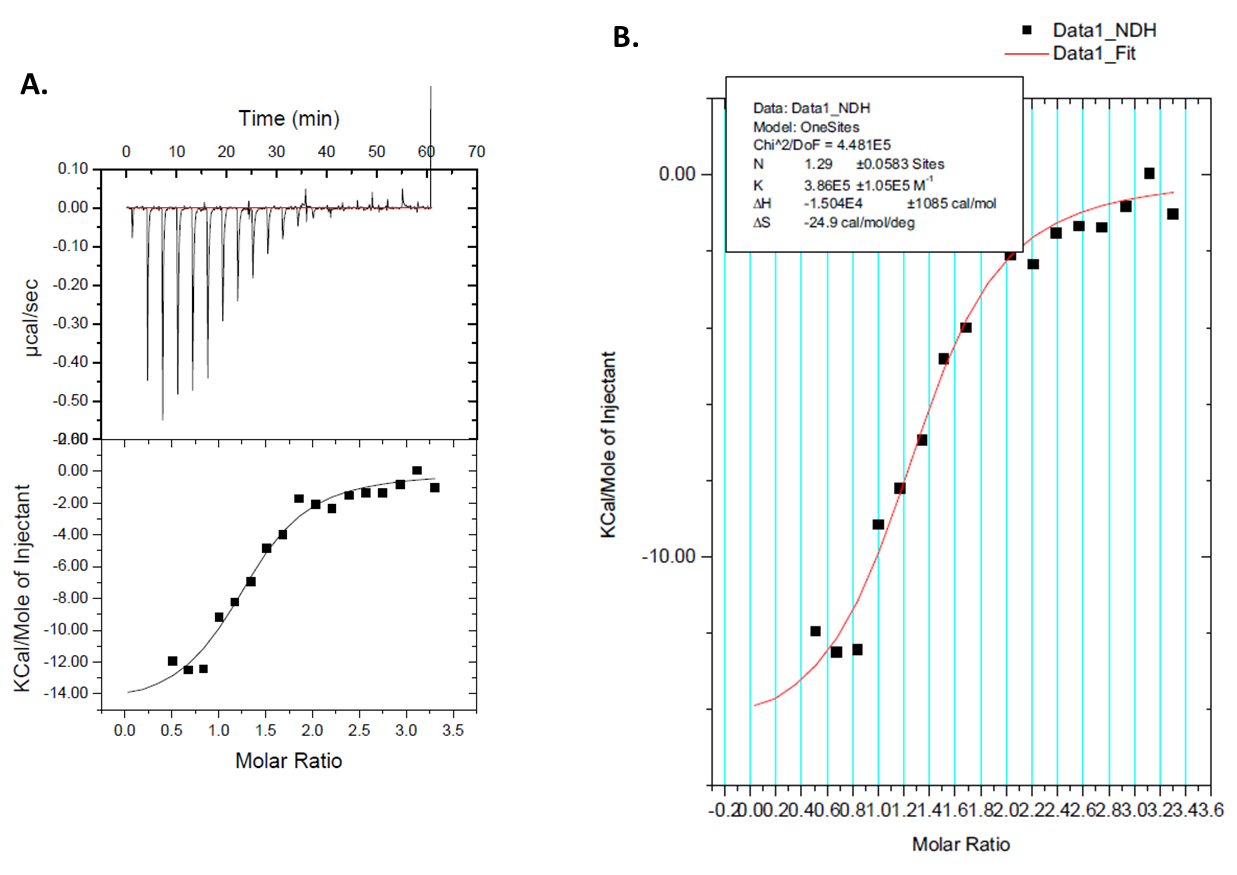
**

**Figure S7.** A.) ITC injection profiles and calculated heat of injectant (same as in the main text). B.) One-site model fit to the ITC titration profile. Enthalpy and entropy changes, the stoichiometry and dissociation constants are indicated in the legend. The stichometry of 1:1.3 likely results from residual MAX:MAX dimers in solution under the ITC conditions.

**Figure S8.** Negative control ITC experiment of MAX injections into buffer. The spikes reflect the heat of dilution.

**References**

1. Che, K.; Kress, T.; Górka, M.; Żerko, S.; Kozminski, W.; Kurzbach, D., Coupled MD simulations and NMR reveal that the intrinsically disordered domain of the breast-cancer susceptibility 1 protein (BRCA1) binds head-on to DNA double-strand ends. *Journal of Magnetic Resonance Open* **2022,** *12-13*, 100069.

2. Epasto, L. M.; Che, K.; Kozak, F.; Selimovic, A.; Kaderavek, P.; Kurzbach, D., Toward protein NMR at physiological concentrations by hyperpolarized water-Finding and mapping uncharted conformational spaces. *Sci Adv* **2022,** *8* (31), eabq5179.

3. Fieber, W.; Schneider, M. L.; Matt, T.; Kräutler, B.; Konrat, R.; Bister, K., Structure, function, and dynamics of the dimerization and DNA-binding domain of oncogenic transcription factor v-Myc11. *Journal of Molecular Biology* **2001,** *307* (5), 1395-1410.

4. Delaglio, F.; Grzesiek, S.; Vuister, G. W.; Zhu, G.; Pfeifer, J.; Bax, A., NMRPipe: A multidimensional spectral processing system based on UNIX pipes. *Journal of Biomolecular NMR* **1995,** *6* (3), 277-293.

5. Lee, W.; Tonelli, M.; Markley, J. L., NMRFAM-SPARKY: Enhanced software for biomolecular NMR spectroscopy. *Bioinformatics* **2015,** *31* (8), 1325-1327.

6. Kizilsavas, G.; Ledolter, K.; Kurzbach, D., Hydrophobic Collapse of the Intrinsically Disordered Transcription Factor Myc Associated Factor X. *Biochemistry* **2017,** *56* (40), 5365-5372.

7. Gorka, M.; Zerko, S.; Konrat, R.; Kozminski, W.; Kurzbach, D., (1)H, (13)C and (15)N backbone resonance assignment of BRCA1 fragment 219-504. *Biomol NMR Assign* **2020,** *14* (2), 289-293.

8. Bergmann, A.; Fritz, G.; Glatter, O., Solving the generalized indirect Fourier transformation (GIFT) by Boltzmann simplex simulated annealing (BSSA). *J Appl Crystallogr* **2000,** *33*, 1212-1216.
